# The Clinical, Philosophical, Evolutionary and Mathematical Machinery of Consciousness: An Analytic Dissection of the Field Theories and a Consilience of Ideas

**DOI:** 10.7759/cureus.12139

**Published:** 2020-12-18

**Authors:** Hassan Kesserwani

**Affiliations:** 1 Neurology, Flowers Medical Group, Dothan, USA

**Keywords:** disorders of consciousness, anatomy & physiology, brain anatomy, philosophy

## Abstract

The Cartesian model of mind-body dualism concurs with religious traditions. However, science has supplanted this idea with an energy-matter theory of consciousness, where matter is equivalent to the body and energy replaces the mind or soul. This equivalency is analogous to the concept of the interchange of mass and energy as expressed by Einstein’s famous equation $E=mc^{2}$. Immanuel Kant, in his Critique of Pure Reason, provided the intellectual and theoretical framework for a theory of mind or consciousness.

Any theory of consciousness must include the fact that a conscious entity, as far as is known, is a wet biological medium (the brain), of stupendously high entropy. This organ or entity generates a field that must account for the "binding problem", which we will define. This proposed field, the conscious electro-magnetic information (CEMI) field, also has physical properties, which we will outline.

We will also demonstrate the seamless transition of the Kantian philosophy of the *a priori* conception of space and time, the organs of perception and conception, into the CEMI field of consciousness. We will explore the concept of the CEMI field and its neurophysiological correlates, and in particular, synchronous and coherent gamma oscillations of various neuronal ensembles, as in William J Freeman's experiments in the early 1970s with olfactory perception in rabbits.

The expansion of the temporo-parietal-occipital (TPO) cortex in hominid evolution epitomizes metaphorical and abstract thinking. This area of the cortex, with synchronous thalamo-cortical oscillations has the best fit for a minimal neural correlate of consciousness. Our field theory shifts consciousness from an abstract idea to a tangible energy with defined properties and a mathematical framework.

Even further, it is not a coincidence that the cerebral cortex is very thin with respect to the diameter of the brain. This is in keeping with its fantastically high entropy, as we see in the event horizon of a black hole and the conformal field theory/anti-de Sitter (CFT/ADS) holographic model of the universe.

We adumbrate the uniqueness of consciousness of an advanced biological system such as the human brain and draw insight from Avicenna’s gendanken, floating man thought experiment. The multi-system high volume afferentation of a biological wet system honed after millions of years of evolution, its high entropy, and the CEMI field variation inducing currents in motor output pathways are proposed to spark the seeds of consciousness.

We will also review Karl Friston's free energy principle, the concept of belief-update in a Bayesian inference framework, the minimization of the divergence of prior and posterior probability distributions, and the entropy of the brain. We will streamline these highly technical papers, which view consciousness as a minimization principle akin to Hilbert's action in deriving Einstein's field equation or Feynman's sum of histories in quantum mechanics. Consciousness here is interpreted as flow of probability densities on a Riemmanian manifold, where the gradient of ascent on this manifold across contour lines determines the magnitude of perception or the degree of update of the belief-system in a Bayesian inference model.

Finally, the science of consciousness has transcended metaphysics and its study is now rooted in the latest advances of neurophysiology, neuro-radiology under the aegis of mathematics.

## Introduction and background

Kantian philosophy

We begin our journey by defining sensation as the awareness of a stimulus and perception as the integration of a sensation into awareness of a specific object or entity. Knowledge is the transformation of sensation into perception. John Locke in 1869, in his Essay of Human Understanding, applied the inductive methods of Francis Bacon. Knowledge comes from the senses and from experience [1].

We begin with a clean slate, a "tabula rasa". Sensation begets memory and memory begets ideas. The dogma is

 

$$sensation\rightarrow memory\rightarrow idea$$

Paraphrasing, knowledge is derived from the senses. Since knowledge comes from sensation, then absolute knowledge is impossible. But sensation can only come from matter, therefore thought is an extension of matter. This was a materialistic philosophy that was shattered with a stroke of brilliant insight by the Irish Bishop, George Berkeley, who sagaciously argued that matter is nothing but a bundle of sensations. If there is no sensation, there is no matter. Matter is a condition of the mind and the only reality is mind. Berkeley had destroyed matter [2].

Enter David Hume, who argued in A Treatise of Human Nature, that ideas, memories, perceptions and feelings are the mind. There is no soul. Hume had destroyed the soul [3]. The adage was "no matter, never mind". Into this conflagration, Immanuel Kant unleashed his ideas and philosophy like a thunderbolt.

So how does knowledge arise? In Immanuel Kant’s Critique of Pure Reason, published in 1781, ideas such as right or wrong, the categorical imperative, and the creator are inherent in the mind and innate, prior to experience. By pure is meant knowledge acquired by the very nature and structure of the mind, in the absence of sensation and experience. This is *a priori* knowledge independent of sensation and experience. Here we have absolute truth and science, through deductive reasoning and buttressed by axioms, a mathematical edifice. $2\times 2=4$ is an absolute truth, independent of time and experience, in this or another alternate cosmos. Here the dogma or paradigm is different, where the innate parameters of space and time coordinate and manipulate the sensations, the "esthetic", and mould them into perceptions and thoughts, the "logic" [4].

How does the mind convert a sensation into a perception and knowledge? In this framework, space and time are the *a priori* organs of perception, the modes of perception, selecting and stratifying stimuli. They are the mind, exercising the laws of mathematics, absolute and necessary. The sensations consolidated into perceptions are henceforth consolidated into conceptions. This is the organ of the mind, the ability to coordinate experience, under the aegis of space and time. Our new dogma is

 

$$sensation\rightarrow perception\rightarrow conception$$

Kant says perception without conception is blind. This absolute truth which arises from the structured ordering of sensations into conceptions via the immutable laws of space and time impressed by the mind is science. So we have the thing-in-itself, the very essence of matter, and the phenomenon (sensation, perception, and conception). We can have an idea of the moon itself through a lower fidelity abstraction of a bundle of corrupt sensations. Below is a summary of these concepts (Table 1) [5].

**Table 1:** Dichotomy between Kantian philosophy and the rest, as exemplified by Hume.

KANT	HUME
A priori	Tabula rasa
Time and space are modes of perception	Experience builds on knowledge
Deductive	Inductive

The binding problem and the integration of information

Critical to any understanding of consciousness is the concept of binding. This is best illustrated with an example. In perceiving an elephant, the very idea of an elephant lies in integrating its contours, geometry, parts, color, and if necessary its texture and sound. These sensations lie in various regions of the cerebral cortex. The organizing mind has to link instantaneously active, seemingly disparate parts of the brain and come up with the percept of an elephant. This is no easy task. However, a field theory where electromagnetic field waves with relevant information from different loci interfere constructively, and unnecessary information from other loci will lead to destructive interference, is an instantaneous and highly efficient mechanism [6].

**Figure 1:**
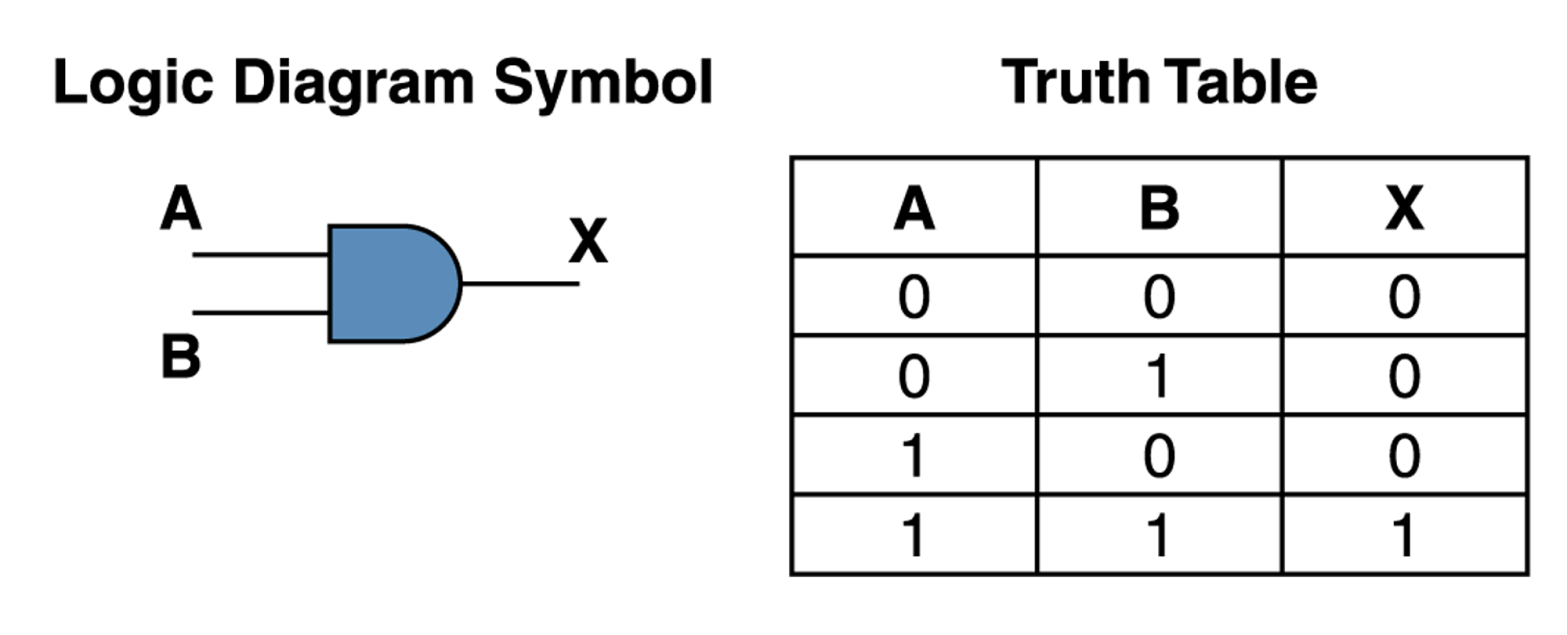
Logic diagram and truth table for an AND logic gate.

The second concept is one of integration of information in the time domain versus the space domain. In the time domain, output of information depends on the input of information in time. Here we have a causal chain of operations in time, or a catena of events cascading through time. Once the information is processed, the system is ready to accept new bits of information. Hence our data is not locked in space, only in time. A good example are logic gates, the simplest being an AND gate, with the rule

$$X=A.B$$

and the truth table and logic diagram (Figure 1) [7].

This is also how synapses work, with temporal integration of information.

**Figure 2:**
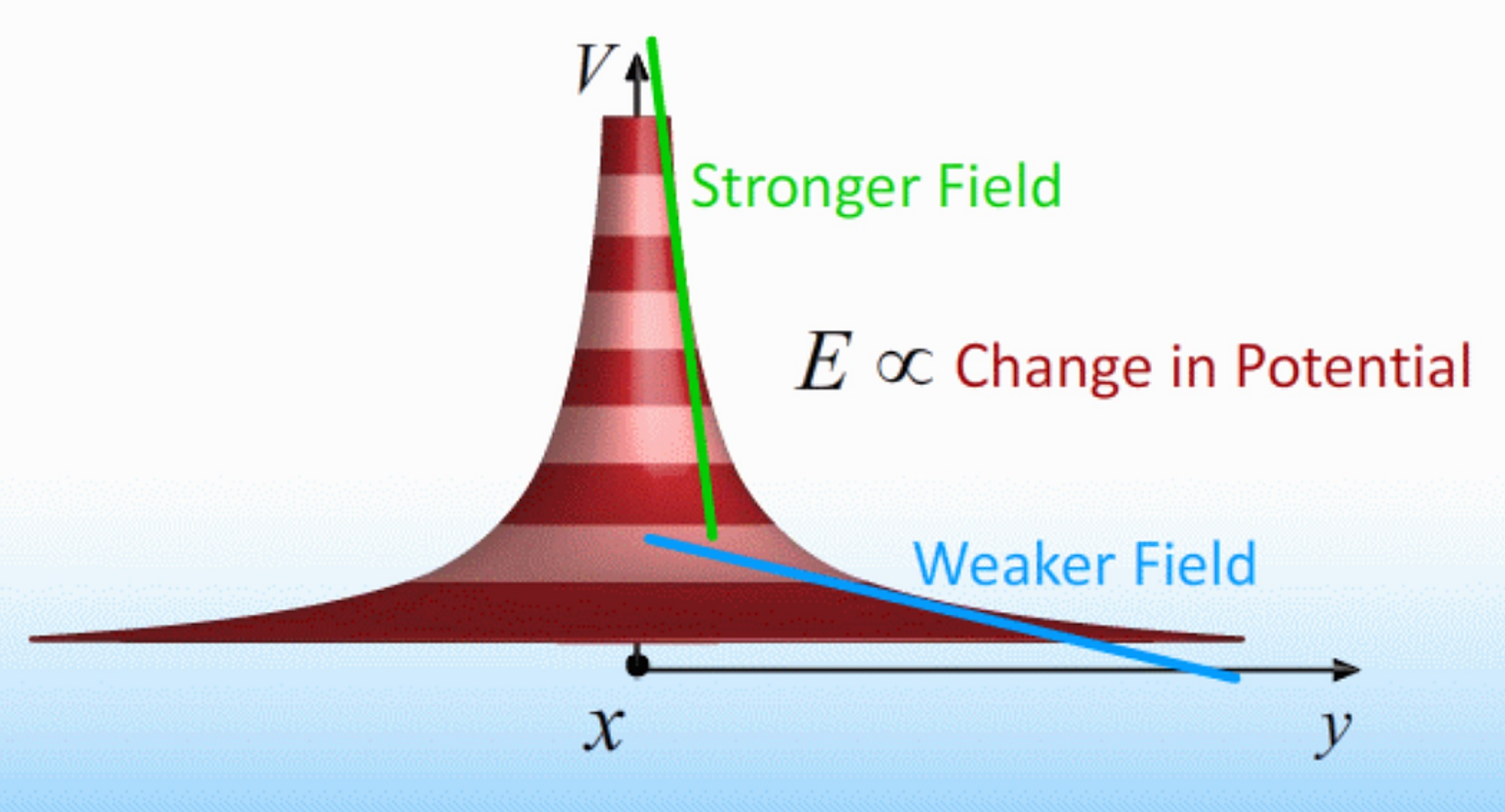
Inverse relation between potential on ordinate axis and distance from source of charge on the abscissa. Voltage (V), potential energy (E) and displacement from source, x and y.

However, the integration of information in space is very different. This is where field theory comes in. A great example is gravity, where the inverse-square law of force is known to most high school students. Here the potential energy of the gravitation field depends on the inverse distance from the center of the Earth, or the height above the surface. The same holds for an electric field potential, where the electric field potential varies inversely with distance away from point source [8].

$$E=-\frac{dV}{dx}=-\triangledown \varnothing$$

where $V$ is the potential difference, $x$ is the distance from the source, and $\phi$ is the field potential. The dependence on space is demonstrated graphically below (Figure 2).

The inverse-law of the electric potential means that the volume of conduction of a single firing neuron has a radius of 80 micrometers and envelopes about 200 neurons. The generation of electroencephalography (EEG) potentials involves the summation of excitatory post-synaptic potentials (EPSPs) and inhibitory post-synaptic potentials (IPSPs) in layer V of the cerebral cortex. The sign of the signal, positive or negative, depends on the afference, whether cortico-cortical or thalamo-cortical. Summation of pyramidal dipoles by synchronous neural activity produces a larger signal [9,10].

Information processing is executed in pathways running in parallel or in series. These two modes of integration are deployed for different purposes (Table 2) [11].

**Table 2:** The dichotomy of in-parallel and in-series processing of information in the brain.

IN-PARALLEL-PROCESSING	IN-SERIES-PROCESSING
Unconscious mind	Conscious mind
Digital	Electro-magnetic field
Automatic action, like riding a bicycle	Learning new information, slower

If electromagnetic wave processing was to occur in parallel, then there is potential for destructive interference of waves. Instead, target neurons are selected and enforced in-series by Hebbian mechanisms, such as long-term-potentiation [11].

As argued by John von Neumann in his powerful little book, The Computer and the Brain, neurons are slow at computation, with a clock frequency of 100 basic operations per second, compared to a small computer that can perform 100 million operations per second. Furthermore, a neuron can compute to an accuracy of two decimal places, whereby a digital device can easily attain an accuracy of 12 decimal places. This lack of logical depth is easily overcome by logical breadth with a vast parallel path network. With $10^{14}$ synapses, the clock frequency skyrockets to $10^{16}$ operations per second [12]. 

Armed with this basic information, we will tackle the concept of the conscious electro-magnetic information (CEMI) field theory and advance it further by exploring the concepts of entropy, information theory, and the surface area-dependent property of human brains and its analogy to black holes. We will attempt to explain what it is that sets apart human brains, a wet medium, from a supercomputer without consciousness by addressing insights from the 10th-century polymath Avicenna's floating-man thought experiment. The tremendous expansion of the temporo-parietal-occipital cortices in the hominid lineage, with its metaphorical and abstract thinking capacity, will be explored as a potential substrate of the minimal neural correlate of consciousness, the so-called posterior "hot zone". Finally, we will outline Karl Friston's free energy principle and belief-updating of prior probability distributions as a gradient flow on a Riemannian manifold.

## Review

Avicenna's floating-man gendaken and sensory deprivation

Prior to Rene Descartes' "Cogito ergo sum" argument for the existence of the self, the 10th-century polymath Avicenna, in his Cannon of Medicine postulated the gendaken, the floating-man hypothesis. In this thought experiment, a man is suspended in air, a floating medium, without any sensory perception, sight, sound, smell or any other form of sensation. Avicenna posits that such a state defaults the individual to a sense of self, or more aptly paraphrased "knowledge by presence" [13]. We feel that this gendaken is a metaphysical argument. Avicenna may have implied the absence of gravity, without actually referring to the concept of gravity. We extend his idea further and refute his claim of "knowledge by presence", by floating the man or woman in a medium with absence of gravity and total sensory deprivation, total deafferentation that includes visceral sensation and all olfactory and thalamic input, including kinesthetic sense. In this state, we claim that sentience, the minimal ability to experience a qualia, the very quality or essence of sensation, the formation of a sensory percept, is impossible. Put simply, it is impossible to experience, to be aware, to be cognizant, without sensory input. This is not a far-fetched idea. As an example, we cite the example of forming a visual percept. In a review article on the biophysics of vision, the author summarized the concept of the formation of a visual percept. To form a visual percept, prior to cognizance, the brain convolves a stimulus, in this case, the function of light intensity, with a filter function, the selection of an ensemble of neurons [14]. The very essence of percept formation involves afferentation, a stimulus. Our dogma is

$$stimulus\rightarrow percept\rightarrow consciousness$$

Freeman's mass action and wave amplitude modulation

**Figure 3:**
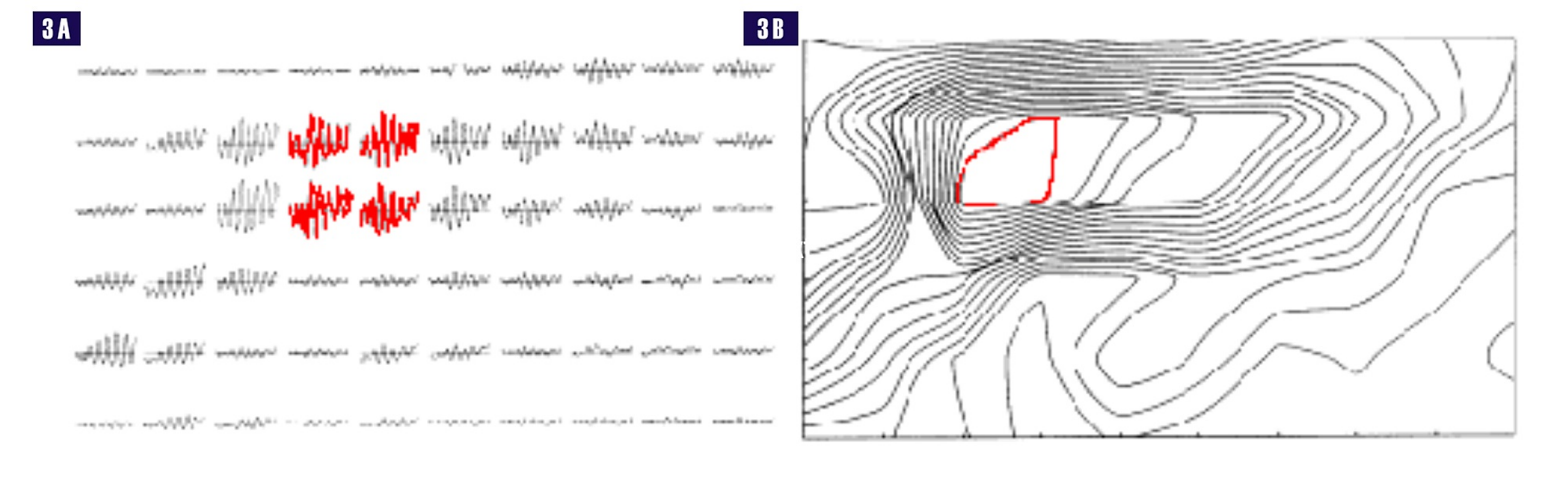
Contour spatial map reflecting amplitude modulation of scent perception. 3A- High frequency gamma bursts at each electrode of grid, highest amplitude (red). 3B- Contour spatial map reflecting amplitude modulation, highest amplitude contour (red).

Walter J Freeman, in a series of stunning experiments in the 1970s, performed electro-corticograhic (ECoG) recordings from the olfactory bulbs and olfactory cortices of rabbits using an $8\times 8=64$ grid, after stimulating the nasal mucosa with the scent of bananas and saw-dust. He was able to establish a high amplitude high-frequency gamma burst, approximately 40 Hertz (Hz), riding on a slow delta (less than 4 Hz) background, the carrier wave, with the perception of the scent. This pattern was visible on all the electrodes of the grid with scent perception. The shape of the carrier wave, the delta wave, did not determine the scent. The pattern of amplitude variation across the cortex determined the perception of the scent. These patterns resembled a topographic map, as seen with the isobars of a weather system, or the plots of elevation of the land on a geographical contour map (Figure 3) [15].

**Figure 4:**
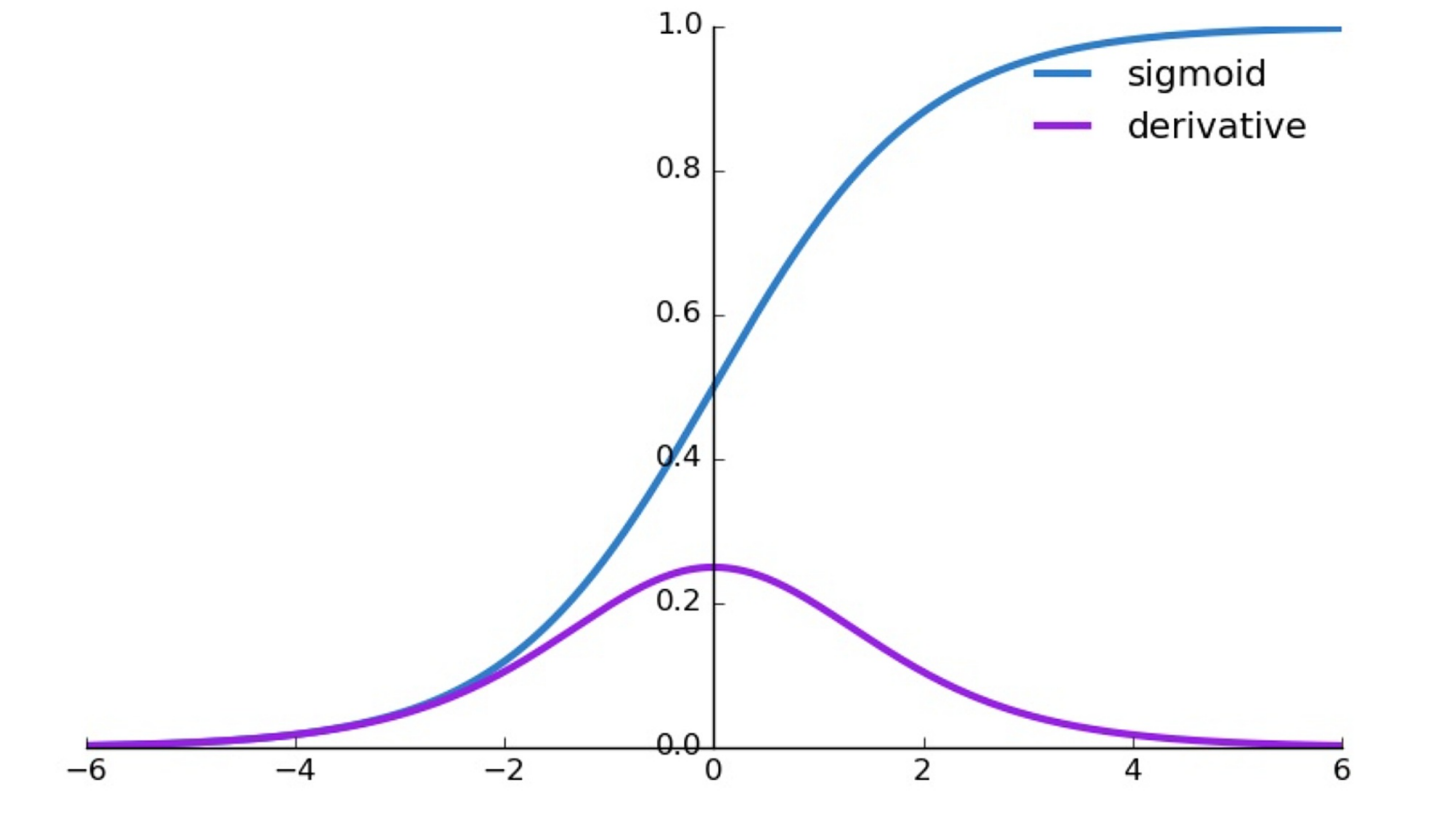
Sigmoid function: relationship between amplitude of a sensory response, ordinate axis, and neuronal firing frequency, abscissa. The derivative of a sigmoid function is a normal or Gaussian distribution. The units are arbitrary.

Another interesting finding which is reflective of all sensory systems in the animal kingdom is the sigmoid relationship between the response of a sensory system and stimulus intensity. This is expressed as a sigmoid relationship between the firing frequency of a neuron (proportional to stimulus intensity) and the response (electroencephalography amplitude, or wave density) (Figure 4) [15,16].

The derivative of a sigmoid function is a Gaussian distribution, a bell-shaped curve, a fact that we will deploy when we look at conjugate priors with Bayesian inference models. In fact, this is easy to prove mathematically, if $y=\frac{1}{1+e^{-x}}$, then by simple calculus

$$\frac{dy}{dx}=y(1-y)$$

Bits, information theory and entropy

We measure information, $I(p)$, by observing the occurrence of an event with probability, $p$. If an event has a probability of $1$, we obtain no information from the occurrence of the event, that is, $I(1)=0$. If two events, with probabilities $p_{1},p_{2}$ occur independently, then the information we obtain from both events occurring will be

$$I(p_{1}*p_{2})=I(p_{1})+I(p_{2})$$

the sum of both informations. These two axioms have the properties of logarithmic functions (Appendix I, II).

Extending this further and by the law of mathematical induction we get

$$I(p^{n}) = nI(p)$$

Since the probability, $p$, lies between $0$ and $1$, inclusively, we can then define information $I(p))$ as

$$I(p))=-log_{2}p$$

The unit of $log_{2}p$ is the bit. A simple example is the flip of a coin, where the outcome is a head or tail. We get

$$I(p)= - log_{2}(1/2) =1$$

which is $1$ bit of information. Flipping the coin $n$ times, with probability $1/2$ for head or tail, yields

$$I(p^{n})=-log_{2}(1/2^{n})=n$$

which is $n$ bits of information. 

Having defined the bit, we can now segue to the concept of entropy in information theory. If we observe a run of $N$ observations with probabilities of $p_{i}$, we obtain $Np_{i}$ observations. For $N$ independent observations, the total amount of information is 

$$I = -\sum_{i=1}^{N}Np_{i}log(p_{i})$$

The average information per event is

$$I /N= -\frac{1}{N}\sum_{i=1}^{N}Np_{i}log(p_{i})=-\sum_{i=1}^{N}p_{i}log(p_{i}) = S$$

This tells us that the entropy $S$ is the expected value of the information of a distribution, expressed as

$$S = &lt; I(p)>$$

which is similar to an expectation value of a discrete distribution, {$f_{i}$} =$F$

$$&lt; F> =\sum_{i=1}^{N}f_{i}p_{i}$$

A succinct and gentle review can be obtained from John R Pierce's book "An Introduction to Information Theory" [17].

Entropy is a measure of the number of ways a system can be configured or the number of states of a system. High entropy means a large number of possible configurations and low entropy means a low number of possible configurations. It is difficult to define a brain state but one can think of the number of ways that a given locus may be activated either in isolation or as part of a connected system, when a brain is engaged in a task. This is a measure of mental flexibility and adaptability of the brain. This is usually measured in time, and brain entropy can be seen as a measure of variability over time. Two commonly deployed surrogate markers are the complexity of an electroencephalographic (EEG) trace. The signal is filtered, and background noise removed, de-noised. The various frequencies are isolated by wavelet decomposition. The spectral density of the waveforms computed by Fourier analysis, and the complexity of the frequency spectrum measured. The analysis can be carried over a time interval of milliseconds [18]. Another technique is functional magnetic resonance imaging (fMRI) blood-oxygen-level dependent (BOLD) signal. The BOLD signal reflects blood flow and implicitly mental activity within a voxel. Here the analysis can be carried out over an interval of seconds. The entropy is calculated by measuring the variability or unpredictability of the BOLD signal over time, while a brain is engaged in a mental task. Indeed, intelligence as measured by vocabulary and reasoning in a study of 892 healthy adults, showed a positive correlation with entropy, as measured by fMRI-BOLD signal variability, in areas of the brain involved with cognitive processes, the pre-frontal cortex, inferior temporal lobes, and the cerebellum [19]. 

An interesting hypothesis of criticality posits that consciousness of the human brain may have started its origins in a primary or primitive state known as the "psychedelic state". This was thought to be a state of high entropy, where the connectivity repertoire of the brain was high and disordered. A crude but apt analogy would be the thermal agitated state of a gaseous medium versus the low entropy state of a crystalline solid. The transition between disorder and order is known as the threshold of criticality, from the psychedelic disordered state to the normal awake and conscious state. This transition is paralleled by a fall in entropy and the emergence of power scaling laws. Psychedelics, such as psilocybin, are serotonin type 2A (5-HT2A) receptor agonists, where these receptors are highly concentrated in the posterior cingulate cortex (PCC). The PCC along with the default mode network (DMN), including the inferior parietal lobule, PCC and pre-frontal cortex, are involved in meta-cognitive functions such as self-reflection, theory of mind and mental time travel, cognitive modalities associated with an awake conscious state. By injecting healthy volunteers with psilocybin, and measuring entropy with the fMRI-BOLD, there was a collapse of organized activity in the DMN [20].

In another study of 1049 healthy volunteers with the fMRI-BOLD technique to measure entropy, there was a sharp dichotomy with high entropy in the cerebral cortex and low entropy in the rest of the brain [21].

Brain entropy also correlated with measures of divergent thinking, such as creativity, fluency, flexibility and originality. A positive correlation was measured for the left dorsolateral prefrontal cortex, pre-supplementary motor area, left dorsal anterior cingular cortex and creativity. A positive correlation was detected for fluency, flexibility and originality for the left inferior frontal gyrus and left middle temporal gyrus [22].

Compared with healthy controls, an fMRI-BOLD entropy measure in 43 schizophrenic patients showed a selective decrease in entropy in the right middle pre-frontal cortex, bilateral thalamus, bilateral caudate and right hippocampus, areas involved with cognition and planning. However, intriguingly, there was increased entropy in areas of the brain associated with visual imagery and hallucinations, such as the right fusiform gyrus, left lingual gyrus, left pre-cuneus, and right superior occipital gyrus [23]. 

High entropy, black holes, and human brains - "the area law"

Traditional and contemporary computers and digital devices have an in-built von Neumann architecture, which have separate processing/computing units and memory storage units. Instead, in the human brain, logical operations and data storage are carried out by synapses in the cerebral cortex, in the same unit. Recently, a two-dimensional material, molybdenum disulfide, may provide a dual logical and data storage platform. This is known as a logic-in-memory device [24]. This two-dimensional logic-in-memory or more precisely data-storage surface is not a mere coincidence but a fundamental property of nature.

Returning to the cerebral cortex, if we think of the human brain as a sphere, the average cerebral cortex measures two-millimeters in thickness, with the radius of the brain at $670$ millimeters (mm). The ratio of thickness of cortex to diameter of the brain is $2/1340 = 0.000149$, rendering the cerebral cortex a simulacrum of a two-dimensional surface [25]. 

With $10^{14}$ synapses and $4.7$ bits of information at each synapse, the human brain can potentially store approximately 2.5 petabytes of information. This stupendous amount of information is in the range of data storage in a super-computer and black holes [26]. 

Traditional concepts of storage of information are via Hebbian mechanisms. Spike-timing-dependent plasticity (STDP) is the basis of Hebbian learning at the synapse between two neurons. The adage is "fire together, wire together". There is antecedent firing and causality; cause and then effect, hence the "timing" in STDP. This provided us with an activity dependent mechanism for plasticity. At the cellular level, long-term potentiation (LTP), with activation of N-methyl-D-aspartate (NMDA) receptors is the molecular basis. The role of long-term depression (LTD) is also important. Time delay between the pre-synaptic stimulus and post-synaptic spike determines whether LTP or LTD is induced, with LTD occurring when pre-synaptic stimulus immediately follows post-synaptic spike [27,28].

**Figure 5:**
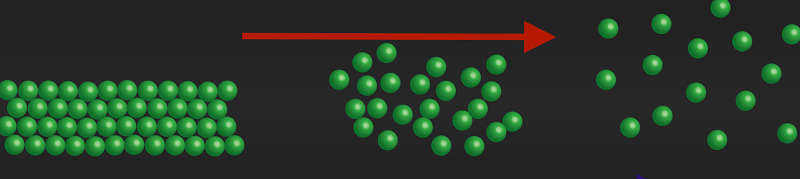
increasing entropy (red arrow) as we move from a solid to liquid to a gas.

Thermodynamic entropy, which is proportional to the number of states in a system, is also expressed as 

$$S= kln(W)$$

where $k$ is the Boltzmann constant and $W$ is the number of potential micro-states. It is well known that the degree of disorder or number of micro-states in a fixed space is proportional to the volume of the space (Figure 5).

However, this volume entropy law changes drastically when we move to a black hole. This volume law changes to an area entropy law. In a black hole

$$S=\frac{\pi Kc^{3}A}{2hG}$$

where $h$ is Planck's constant, $k$ is a constant, $G$ is the Gravitational constant, $c$ is the speed of light and $A$ is the area of the event horizon [29]. One can see that

$$S\propto A$$

Motivated by these similarities between the brain and black holes with respect to memory storage, Dvali constructed an artificial quantum neural network in a $d$-dimensional space and showed that this network possessed a $d-1$ dimensional surface whose micro-state entropy magnitude obeyed the area law [30]. 

The high entropy of the human brain with a potential abeyance of the area law of entropy concurs with Karl Pribram's holonomic or holographic theory of brain function [14].

Frequentist versus Bayesian inference models of decision making

Given a prior distribution $p(x)$ and some set of evidence E, the posterior distribution on $x$ is $p(x/E)$, and is expressed as

$$p(x/E) = \frac{p(E/x)}{p(E)}p(x)$$

The posterior probability $p(x/E)$ and the prior probability $p(E/x)$ distributions belong to the same family of probability distributions . $\frac{p(x)}{p(E)}$ is the likelihood ratio and is a conjugate prior to the posterior probability distribution.

Statistical inference is divided into two schools of thoughts, the frequency approach, which is empirical and depends on direct observations with a number of trials, and direct computation of confidence intervals and tests of significance. The Bayesian inference approach infers the existence of a prior probability, a continuous distribution, which is difficult to calculate. Below is a summary of the difference between the frequentist and Bayesian approach (Table 3) [31].

**Table 3:** Comparison of models of decision making by the brain: frequentist versus Bayesian inference

FREQUENTIST APPROACH	BAYESIAN APPROACH
Binomial and normal distribution	Beta distribution
Significance tests and confidence intervals	Likelihood ratios
No prior probabilty	Prior probability
Concurs with an empirical philosophy; inductive reasoning	Concurs with Kantian philosophy; deductive reasoning

The updated posterior probability distribution is in the same family as the prior probability distribution. However, the updated or posterior probability distribution is a conjugate prior to the observation distribution. The beta probability distribution is a conjugate prior to the binomial observation distribution, and the normal distribution is self-conjugate (Appendix III, Table 4) [32].

**Table 4:** Table demonstrating conjugate priors of various observation probability distributions. The prior and posterior distribution probabilities belong to the same family of probability distributions.

OBSERVATION	PRIOR PROBABILITY	POSTERIOR PROBABILITY
Bernoulli / Bernoulli distribution	Beta distribution	Beta distribution
Poisson distribution	Gamma distribution	Gamma distribution
Normal distribution	Normal distribution	Normal distribution
Normal distribution	Gamma distribution	Gamma distribution

The variational free energy principle, the Fokker-Planck equation, and Kullback-Leibler divergence

The theory here is to compute a calculus of belief based upon probability distributions. The human brain acts as a comparator constantly editing inputs such as sensations and perceptions with internal models of the outside world. In so doing, it reduces this difference and minimizes the so-called "free energy". This free energy contains the element of surprise or entropy. Minimizing the element of surprise minimizes the entropy. The boundary between the outside and the inner world is defined by the Markov blanket, a hypothetical boundary between self and non-self. The variational free energy is defined by a mathematical equation with gradient flows, a field. The field is produced by neuronal activity. The gradient flow is the variational free energy which determines the entropy. The model of the world can be described by the logarithm of the probability of sensations, the gradient flow and potential energy of the field. 

**Figure 6:**
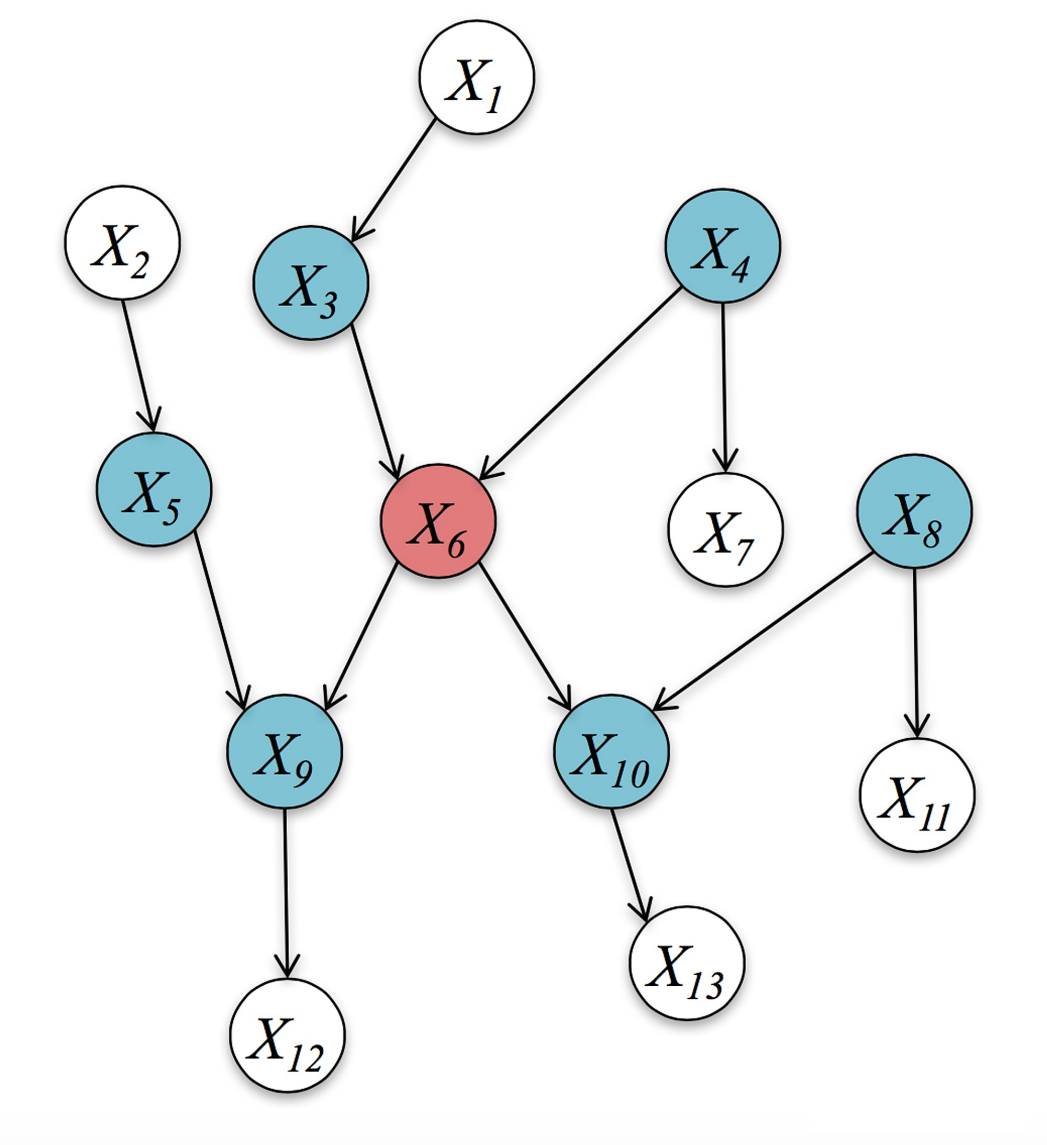
A graph, with edges and nodes, demonstrating the Markov blanket of one red node and blue nodes. The blue nodes are the parents or co-parents of the red node.

Interpreted in terms of Bayesian inference, this is known as belief-updating. We see the data, a logarithmic function and update posterior beliefs with prior beliefs. This adaptable landscape is continually updated with new sensory information. This is the informational geometry where one moves in the space of beliefs. An information geometry is a random dynamic system with internal and external states separated by a Markov blanket; an example is outlined below for a graph (Figure 6). 

The Markov blanket is the minimal set of nodes that separate the $X_{i}'s$ from the rest of the graph. If there is no route between two variables and they share no ancestors, they are independent. The Markov blanket of $X_{6}$ is

$${X_{3},X_{4}, X_{5},X_{8}X_{9},X_{10}}$$

where

$$p(X_{1},......,X_{n})=\prod_{i=1}^{n}p(X_{_{i}}|parents(X_{i}))$$

The intrinsic state is state-based or mechanical and the external state is belief-based. The states are probabilistic and the external state is parametrized by the internal states. A simple example is sensation from the fingers, the sensory input enters the informational geometry, where it is updated. If the gradient of the flow in this geometry is steep, then there is an updating of information. If the gradient is zero, there is no update and no mental action. The curvature of the field determines the steepness of the gradient, corresponding neural activity and mental action. This is known as the "precision of information". The agency of perception, active inference, and sentience is neuronal activity, delimited by the Markov blanket [33]. Psychedelics disrupt this belief-updating system and one may lose one's sense of identity, which is known as depersonalization. Next, we will give this hypothesis a mathematical foothold.

The Fokker-Plank Equation

The Fokker-Plank (F-P) equation describes the time evolution of a probability density function of a stochastic process. It consists of a free energy functional in the space of a probability distribution which is a linear combination of an energy potential and an entropy. The F-P equation describes the gradient flow of this free energy functional on a Riemannian manifold of probability distributions (Appendix IV) [34].

Let us first consider a standard stochastic equation, a forme fruste of the Langevin equation

$$dx=-\triangledown \Psi (x)dt +\sqrt{(\beta )}dW$$

where $\Psi (x)$ is a scalar valued potential function, $\beta$ is a constant and $dW$ is white noise, a fluctuation.

What is the origin of this equation and what does it physically mean? It describes frictional and random forces in a non-equilibrium system. Consider a simple one-dimensional Brownian random system, such as the random motion of particles in a medium of viscosity, $\eta$. Newton's equation of motion for the particle is

$$m\frac{dv}{dt}=F(t)$$

where $m$ is the mass, $x$ is the position, $v$ is the velocity, and $F(t)$ is the total and instantaneous force on the particle at time $t$. 

Assume that we only have a frictional force, $F(t)$. Then by Stoke's law, 

$$F(t)=m\frac{dv}{dt}=-(6\pi \eta a)v$$

where $a$ is the radius of the particle. Solving this first-order differential equation, we obtain

$$v(t)= e^{-\frac{\gamma t}{m}}v(0)$$

where $\gamma =6\pi \eta a$. If this is true then the velocity of the particle should decay to a negligible amount after a long time, which we know is not true because

$$&lt; v^{2}>=\frac{kT}{m}$$

where $k$ is Boltzmann's constant and $T$ is the temperature. Hence $F(t)$ needs to be modified by adding a random or fluctuating force, $\delta F(t)$, as follows

$$m\frac{dv}{dt}=-\gamma t+\delta F(t)$$

This is the Langevin equation, which has two components: a friction force and a fluctuating component known as noise. This is the simplest example of a random system, a one-dimensional Brownian motion. These concepts can be extended into random probability distributions as follows.

The F-P equation describes the time evolution of the probability density function, $p$, of the Langevin equation and describes contour lines

$$\frac{\partial p }{\partial t}=\triangledown .[p\Psi +\beta \triangledown p ]$$

The first term, the divergence of the product of the probability density and the scalar potential function is the "drift term" and the second term, the Laplacian of the probability density, is the "diffusion term" generated by the white noise. 

The free energy functional is a scalar-valued function defined on the space of the probability density function and is defined as

$$F = U-\beta S$$

where $\beta$ is the temperature and $U$ is the potential energy 

$$U=\int p \Psi dx$$

is defined on a state space and $S$ is the entropy

$$S=-\int p log p dx$$

Kullback-Leibler Divergence

The Kullback-Leibler (K-L) divergence between two continuous probability distributions, $p$, and $q$, is defined as the entropy difference between the two distributions [35]:

$$D(p||q)=\int_{-\infty }^{+\infty }p(x)log\frac{p(x))}{q(x)}dx$$

If $p(x)=q(x)$, then $D(p||q)=0$, and no information is lost or gained, noting that the entropy, $S$, is

$$\int_{-\infty }^{+\infty }p(x)log(p(x))dx$$

K-L divergence is not a distance measure or metric, because

$$D(p||q)\neq D(q||p)$$

**Figure 7:**
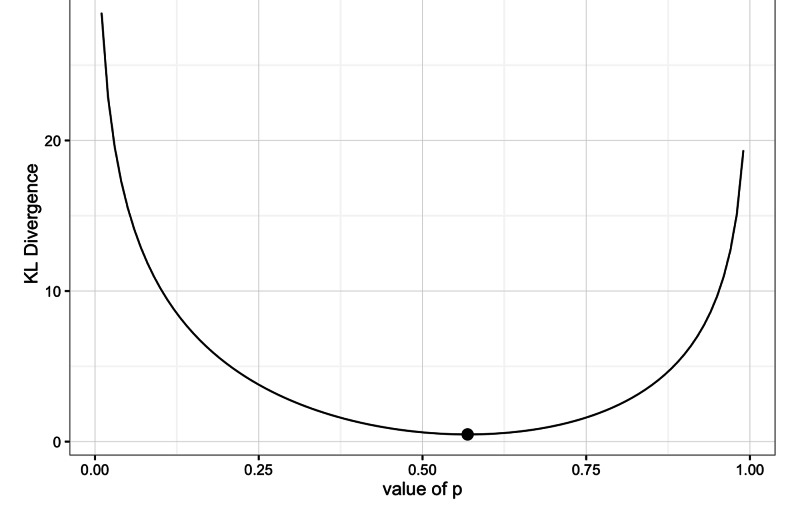
Plot of K-L divergence versus binomial probability distribution (p(x)). Kullback-Leibler (K-L) divergence, probability (p).

If $p(x)$ is a binomial distribution, then a plot of K-L divergence looks like the diagram below (Figure 7).

Armed with the concepts of entropy, Bayesian inference, the F-P equation and K-L divergence, we can now unpack the theory of perception and minimization of the free energy principle. A biological system, like the human brain, has to interpret a sensory input, which follows a discrete or continuous probability distribution based upon an internal dynamic regenerative model, via Bayesian inference. Before embarking on the details, we need a few definitions (Table 5) [36].

**Table 5:** Definitions of parameters for Bayesian inference. Recognition density (q), posterior density (p).

PARAMETER	DEFINITION
surprisal or surprise	negative of the logarithm of the probability of an outcome
recognition density (q)	probability distribution of sensory input
conditional or posterior density (p)	probability distribution of map from sensory data to cause

$q$ depends on $\theta$, the cause of the sensory input, and $\mu$, the internal brain state.

$p$ depends on $\theta$, and the sensory signals and their motion, $s$, and a probabilistic generative model, $m$.

The surprisal, $T$, is expressed as

$$T= -log(p)$$

where $0\leq p\leq 1$. Note that when $p$ is low, then $T$ is low and vice versa.

In simple terms, the brain interprets sensory input based upon an internal representation of the outside world, and this difference is continually updated. The prior and posterior probability distributions are weighed by the likelihood ratio of a Bayesian inference model. 

The free energy of the system can be described in three different ways. 

Classically, the free energy, $F$, is a sum 

$$F= U+\beta S$$

where $U$ is the internal energy of the system, $\beta$ is the temperature, and $S$ is the entropy. 

However, the free energy can be interpreted as a measure of surprise

$$F=&lt; ln(q)> -&lt; ln(q)>$$

where$&lt; >$ is the expectation value.

The free energy can also be interpreted as

$$F=D(q||p)-ln(p)$$

which is the difference between the K-L divergence and surprise. By reducing $D(q||p)$ and $-ln(p)$, noting that $-ln(p) >0$, the free energy $F$ is minimized [37]. 

In summary, belief-updating involves continual updating of both prior and posterior probabilities in a Bayesian inference model, and this reduces the divergence between both probability distributions, which minimizes the free energy, via an action principle, (Appendix V). 

In physics, any vector field can be described by the sum of the gradient of a scalar potential, $\varnothing$, and the curl of a vector potential, $Q$, the Helmoltz decomposition (The Fundamental Theorem of Vector Calculus).

$$\triangledown \varnothing +\triangledown \times Q$$

with the condition that $\triangledown \times \varnothing =0$ and $\triangledown .Q=0$

Setting $L=-log(p)$ is our scalar potential and $\Gamma =\epsilon kT$ as the fluctuation amplitude, where $\epsilon$ is a constant, $k$ is the Boltzmann constant and $T$ is the temperature, we get the F-P operator as a Helmholtz decomposition where 

$$L(x)= -ln(p(x)) ,\triangledown \times L(x)=0$$

and 

$$f(x)=(\Gamma -Q)\triangledown ln(p), \triangledown .f(x)=0$$

where $Q=-Q^{T}$ is an anti-symmetric matrix and $\Gamma$ is a scalar quantity, an identity matrix. 

Why is $Q=-Q^{T}$ an anti-symmetric matrix that is divergence free ? We know that the curl of a vector valued-function $f=\begin{pmatrix} f_{1} &f_{2} & f_{3} \end{pmatrix}$ is 

$$\triangledown \times f=\bigl(\begin{smallmatrix} 0 & -\partial _{3} &\partial _{2} \\ \partial _{3}& 0 & -\partial _{1}\\ -\partial _{2} & \partial _{1} & 0 \end{smallmatrix}\bigr)\bigl(\begin{smallmatrix} f_{1}\\ f_{2}\\ f_{2} \end{smallmatrix}\bigr)=Qf$$

where $Q=-Q^{T}$ is an anti-symmetric matrix, and

$$\triangledown .(\triangledown \times f)=\triangledown .Qf=0$$

A proof of this identity is outlined in Appendix VI.

$L(x)$ is a solution of the F-P equation and $\Gamma \triangledown ln(p(x))$ is a dissipative flow that ascends gradients established by the surprisal element. This is a curl-free flow that neutralizes the dispersion of density caused by the random fluctuations, $\Gamma$. 

$-Q\triangledown ln(p(x))$ is a solenoidal, divergence-free, flow that circulates in contours.

**Figure 8:**
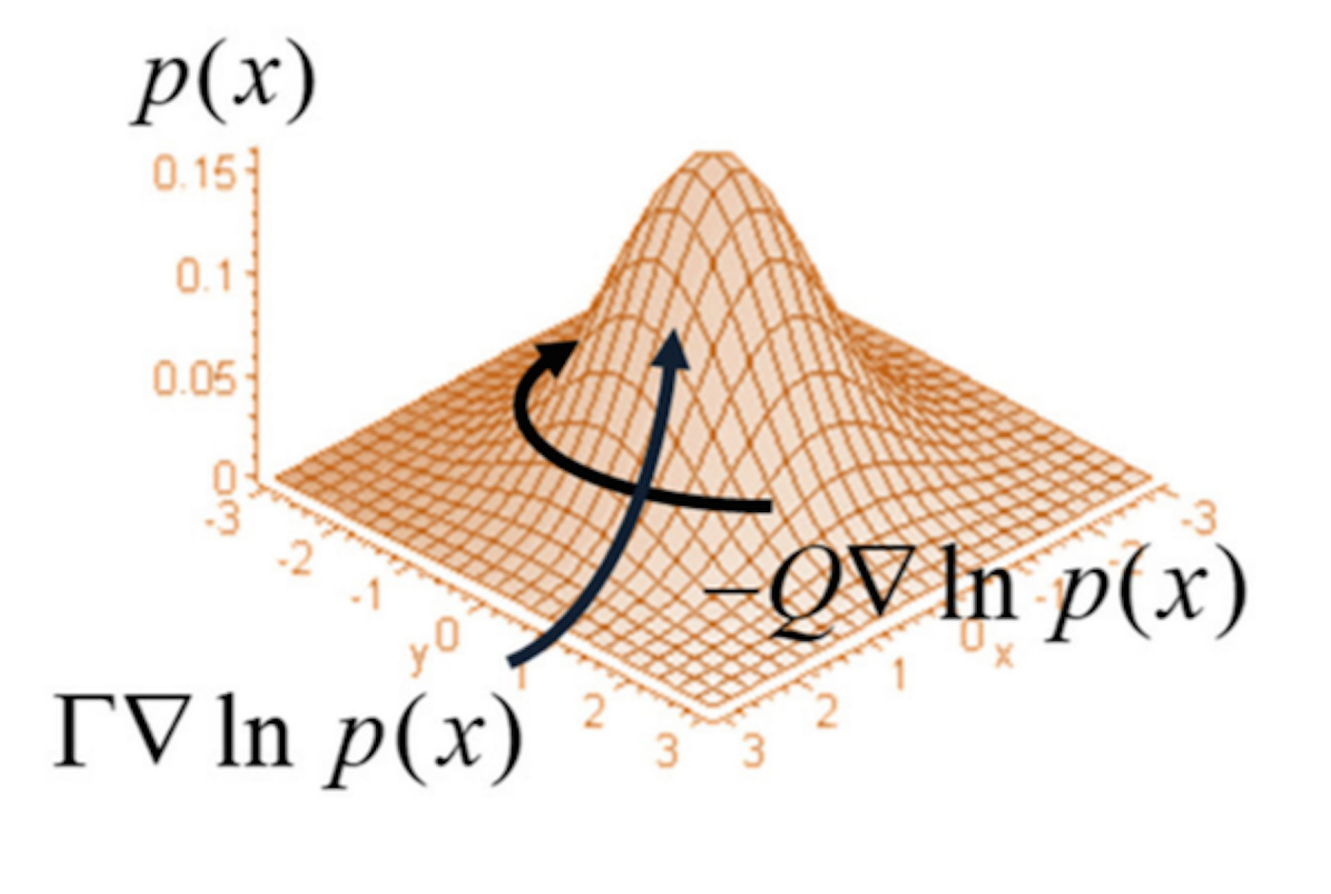
Dynamics of non-equilibrium state between gradient flow, surprisal, and amplitude fluctuations. Probability (p), log to the base e is natural logarithm (ln).

These probability flows occur in a non-equilibrium state. The dynamics of the divergence-free and curl-free flow is demonstrated below (Figure 8) [38].

In Figure 8, if there is no gradient across contour lines, the ascent is shallow, and there is minor belief-updating, with a small surprisal. If the ascent is steep, the surprisal is high and belief updating significant. Where the average values are equal, we obtain a contour line. A flat manifold means no belief update and no interaction with the environment.

Neural correlate of consciousness, the "posterior hot zone" and polymodal abstraction 

The very essence of a sensory perception such as the scent of a flower or the blueness of the sky is known as qualia. In ancient Sanskrit, this is known as "rasa" ; the juice, essence or taste, the core esthetic flavor of a sensation. What are the basic neural circuits that can evoke qualia or rasa? This is a loaded question as it implies that there are privileged loci in the brain that are endowed with the property of transduction of a conscious state? More accurately, one should ask what are the mechanisms that are operational for a privileged or strategic workspace to elicit or evince consciousness? We earlier alluded to the convolution of a visual stimulus with a Gabor filter function, or selective activity of a neuronal ensemble, as the minimal pre-requisite for setting up the perceptual mechanism of a visual scene [14]. We also explored the concept of synchrony of neuronal firing and gamma oscillations in evincing perception and awareness of an olfactory stimulus.

We know that extirpation or traumatic damage of the pre-frontal lobes may alter behavior but preserves sentience. The same holds for the cerebellum; damage or removal may cause incoordination and cognitive slowing but consciousness is preserved [39].

The thalamo-cortical system is a critical pathway for consciousness as thalamic infarcts are known to lead to stupor and absence epilepsy with paroxysmal bursts of three per second paroxysmal epileptiform spike-slow discharges are known to impair consciousness for the duration of the epileptiform discharge. Comatose and anesthetized patients display a dramatic slowing of cerebral activity as measured by electroencephalographic recordings. This is not the case in in the awake state or with rapid-eye-movement (REM) sleep where dreams are vivid, and faster low-voltage activity is more prominent. The integration of activity between thalamo-cortical pathways and the frontal lobes is known to sustain the conscious state with rapid re-entry and sustained activation of multiple parallel distributed cerebral areas [40].

One of the major events to occur in hominid evolution, as early as 3.5 million years ago, is a relative reduction in the volume of primary striate cortex with posterior displacement of the lunar sulcus, which delimits the striate cortex, separating it from the parietal lobe. This was accompanied by an expansion of the inferior parietal lobes and the inferior temporal lobes [41].

Direct electric stimulation of the posterior parietal lobe produces an intention to move and direct right fusiform gyrus stimulation elicits perception of faces. Traumatic damage to the posterior corpus callosum connecting both parietal lobes is found in almost all patients in a persistent vegetative state after one year and predicts an extremely poor outcome. Prognosis with frontal lobe damage is far better. It is well established that bilateral pre-frontal lobe damage with psychosurgery and trauma does not alter conscious awareness [42]. Functional magnetic resonance imaging studies (fMRI) show that bilateral temporo-parietal-occipital connectivity best differentiates patients in a minimally conscious state versus a persistent vegetative state [43]. Memory loading of visual short-term memory correlated better with parietal cortex than with frontal cortex by fMRI studies. The posterior lobes of the brain, the temporo-parietal-occipital lobes, are hence referred to as the "posterior hot zones" [44].

The angular gyrus in the inferior parietal lobule is a major hub for cross-modal abstraction and metaphor representation. It is involved in the interface from perception-to-recognition-to-abstraction with selective and mental manipulation of information. Stimulation of the right angular gyrus leads to an out-of-body experience. Functional imaging studies have shown bilateral activation of the angular gyri in theory-of-mind activity [45].

CEMI field theory

Synchronous firing of neurons in disparate regions of the brain correlates well with conscious perception. Experiments in the 1990s demonstrated that recordings from the visual cortex of monkeys observing approximating versus receding bars revealed synchronous firing of neurons in disparate parts of the brain with zero time lag, when the monkeys become consciously aware of the stimuli. These experiments are akin to binocular rivalry [46,47].

Resonance in the gamma frequency range, 30-50 Hertz (Hz), correlates with visual perception in the visual (V4) area in monkeys trained in generalized flash suppression experiments [48].

Intra- and extra-cellular recordings from nearby neurons in a rat brain slice after application of a weak electric field (EF) induced synchronous in-phase neuronal firing of nearby neurons, without affecting neuronal frequency. Increasing the EF increased the phase-locking amplitude of synchronous neuronal firing [49].

Weakly applied external electric fields were able to entrain local field potentials in ferret visual cortex [50].

Stitching together these observations, the CEMI field is an electromagnetic field induced by an ensemble of neurons that through variation and by Faraday's law of induction may induce downstream currents. This recruitment of neurons depends upon synchronous firing of groups of neurons, some of which are disparate. This model is concordant with Hebbian learning mechanisms, instantaneous, a physical and not a spiritual phenomenon. It is subject to the laws of physics. Being a field theory, with waves, it can be measured, with EEG, fMRI-BOLD and magnetic encephalography (MEG). Furthermore, out-of-phase ensembles can lead to destructive interference and nullification of information, as seen with LTD.

## Conclusions

There is no grand unified theory of consciousness. The CEMI field theory and the free energy principle both have a biophysical basis and/or mathematical framework. They complement each other, but have different premises. The CEMI field theory is based on electromagnetic wave theory and the free energy principle is based on the action principle of physics and probability density flows. Both have clinical, electrophysiological, and radiological evidence to varying degrees. The human brain is a unique structure with a fantastic amount of entropy, which is compartmentalized. This entropy seems to obey the area law of black holes and not the volume law of ordinary matter. The human brain has honed millions of years of biological evolution by convolving poly-afferent sensory inputs with activation of selective ensembles of neurons, with synchronous firing of disparate regions of the brain. The metamorphosis of perception-to-conception seems to involve the synchronous activation of strategic parts of the brain including the posterior thalamo-cortical radiation in unison with activation of the posterior temporo-parietal-occipital lobes. The paleo-neurological and advanced neuro-imaging studies of normal and pathological states are highly supportive of a significant role for this "posterior hot zone". These ideas seem to evolve in parallel to the modern developments of physics, especially that of the action principle, information theory, and entropy. Consciousness is now more than a metaphysical concept; rather a phenomenon whose basis is anchored with the breathtaking advances of neurophysiology, neuroradiology, and the firepower of mathematics.

## Figures and Tables

**Figure 9:**
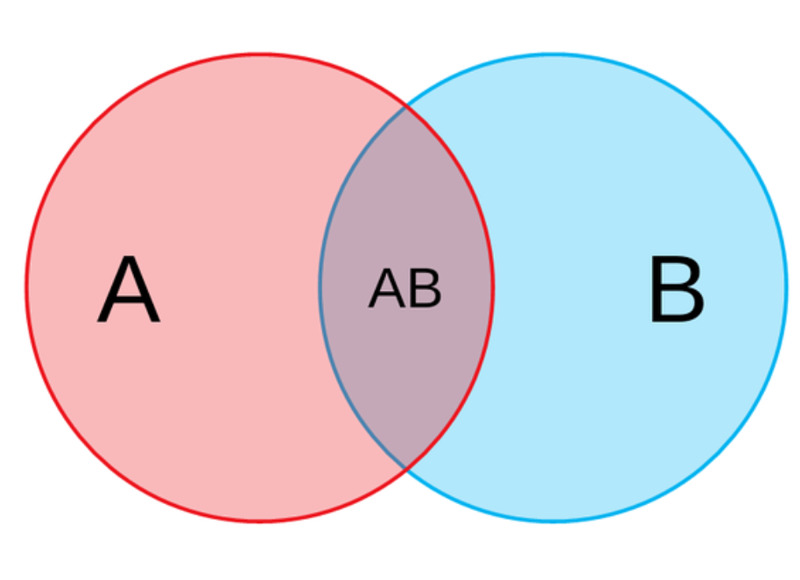
Venn diagram: sets A and B, not mutually exclusive. Both A and B, represented as AB

## Appendices

Appendices

$I$. Derivation of law of entropy via Gibb's inequality

Gibb's Inequality

For $y=lnx$, note that 

$$\frac{d}{dx}(lnx)= \frac{1}{x}$$

The equation of the tangent line at $x=1$ is $y=x-1$, and since $lnx$ is concave downwards, we have for $x> 0$

$$lnx\leq x-1$$

Let $P=\left \{ p_{i} \right \}$ be a discrete probability distribution, the

$H(P)-ln(n)=\sum_{i=1}^{n}p_{i}ln(\frac{1}{p_{i}}) -ln(n)\sum_{i=1}^{n}p_{i}=\sum_{i=1}^{n}p_{i}[ln\frac{1}{p_{i}}]+ln(\frac{1}{n})]=\sum_{i=1}^{n}p_{i}ln(\frac{1/n}{p_{i}})\leq 0$

Therefore,

$$0\leq H(P)\leq ln(n)$$

When only one of the $p_{i}$'s is one, we get $H(P)=0$. When all the probabilities are equal at $\frac{1}{n}$, then we get

$$H(P)=ln(n))= -ln(p_{i})$$

$II$. Conditional probability and Bayesian inference

Let $A$ and $B$ be represent two events and let $P(A/B)$ be the conditional probability of $A$ given $B$, then

$$P(A/B)= P(A\cap B)/P(B)\Rightarrow P(A\cap B)=P(A/B)P(B)$$

and 

$$P(B/A)= P(B\cap A)/P(A)\Rightarrow P(B\cap A)=P(B/A)P(A)$$

and since $P(A\cap B)=P(B\cap A)$, we get 

$$P(A/B)P(B)=P(B/A)P(A)\Rightarrow P(A/B)=P(B/A)P(A)/P(B)$$

This is Bayes's theorem. Paraphrased;

                                                 posterior probability = likelihood ratio $\times$prior probability

where the likelihood ratio is $P(B/A)/P(B)$ and the prior probability is $P(A)$.

The posterior probability is the probability that some hypothesis is true given the evidence. The prior probability is the probability that the hypothesis was true when the evidence was collected. The likelihood is the probability that the evidence was collected if the hypothesis was true. If the observations come from a continuous distribution, the likelihood is defined as the joint probability distribution. The prior probabilities are usually not known and usually introduced as inductive probabilities expressing the degree of belief. The likelihood is a statistical probability.

Two events $A$ and $B$ are independent if

$$P(A/B)=P(A)$$

and

$$P(B/A)=P(B)$$

Then 

$$P(A\cap B)=P(A)P(B)$$

$III$. Bayesian inference and the beta distribution

Suppose an event has an unknown probability $p$ and occurs $x$ times in $n$ trials. Then we ask the question, what is the probability that $p$ lies between two fixed values $a$ and $b$? We first apply the binomial distribution and determine the probability the event occurs $x$ times in $n$ number of trials. This is the likelihood

$$\frac{n!}{x!(n-x)!}p^{x}(1-p)^{n-x}$$

The posterior probability that $p$ lies between $a$ and $b$ is 

$$\frac{\int_{a}^{b}p^{x}(1-p)^{n-x}dp}{\int_{0}^{1}p^{x}(1-p)^{n-x}dp}$$

The factor $\frac{n!}{x!(n-x)!}$ cancels out. Here it is assumed that the prior distribution is a continuous distribution, a rational degree of belief. 

Let us carry out an imaginary experiment whereby we roll a white ball, $w$, along a unit line, that scales from $0$ to $1$. Assume the white ball settles at a point $p$.  Let us then roll a blue ball, $b$, along the same line $n$ times. Let $X$ be the distribution of the number of blue balls that settle to the left of the white ball. Then the probability that $x$ number of blue falls settle to the left of $p$ follows a binomial distribution

$$P(X=x/p)=\binom{n}{x}p^{x}(1-p)^{n-x}$$

The sum of probabilities of all possible $p's$\

$$P(X=x)=\int_{0}^{1}\binom{n}{x}p^{x}(1-p)^{n-x}dp$$

Now through $n$ balls along the unit line and mark the landing sites. Then roll the white ball. Then the white ball has an equal chance of landing to the left of each blue ball. Therefore,

$$P(X=x)=\frac{1}{n+1}$$

We then have 

$$\binom{n}{x}\int_{0}^{1}p^{x}(1-p)^{n-x}dp=\frac{1}{n+1}$$

and

$$\int_{0}^{1}p^{x}(1-p)^{n-x}dp=\frac{x!(n-x)!}{(n+1)!}$$

Let $x=r-1$ and $n-x = s-1$, we obtain the beta function

$$B(r,s)= \int_{0}^{1}p^{r-1}(1-p)^{s-1}=\frac{(r-1)!(s-1)!}{(r+s-1)!}=\frac{\Gamma (r)\Gamma (s)}{\Gamma (r+s)}$$

Next, we will derive the beta probability density function (pdf).

$$P(X=x)=\binom{n}{x}\int_{0}^{1}p^{x}(1-p)^{n-x}dp=\binom{n}{x}B(x+1,n-x+1)$$

and

$$P(a&lt; p&lt; x)|X=x)=\frac{\int_{a}^{b}\binom{n}{x}p^{x}(1-p)^{n-x}dp}{\binom{n}{x}B(x+1,n-x+1)})=\frac{\int_{a}^{b}p^{x}(1-p)^{n-x}dp}{B(x+1,n-x+1)})$$

Therefore, the beta pdf is

$$f(p)=\frac{\Gamma (a+b)}{\Gamma (a)\Gamma (b)}p^{a-1}(1-p)^{b-1}, 0&lt; p&lt; 1$$

$IV$. Riemannian manifold

A Riemannian metric on a smooth manifold, $M$, is a two-tensor field $g\in \mathfrak{T}^{2}(M)$ that is 

*1) *symmetric $g(X,Y)=g(Y,X)$

*2) *positive definite; for $X> 0$, $g(X,X)> 0$

A Riemannian metric determines an inner product on each tangent space $T_{p}M$ such that 

$(X,Y):= g(X,Y)$ for $X,Y\in T_{p}M$.

The length or norm of any tangent vector $X\in T_{p}M$ is 

$$|X|:=&lt; X,X> ^{\frac{1}{2}}$$

$V$. Principle of least action 

We define an action $S[x(t)]$

$$S[x(t)]=\int_{t_{1}}^{t_{2}}dtL(x,\dot{x})$$

where 

$L(x,\dot{x})=T(\dot{x})-V(x)$

and $T(\dot{x})$ is the kinetic energy and $V(x)$ is the potential energy.

The extremal path $\delta S$ is set to $0$ and defined as

$$\delta S=\delta \int_{t_{1}}^{t_{2}}dtL(x,\dot{x})=0$$

$VI$. Curl of grad = 0 and Div of Curl =0

The Levi-Civita symbol is defined as 

$\varepsilon _{ijk}=+1; if (ijk)$ is even; $(123)$, $(231)$,$(312)$\

$\varepsilon _{ijk}=-1$; if $(ijk)$ is odd; $(321)$, $(132)$, $(213)$

$\varepsilon _{ijk}= 0$; if $i=j$, $j=k$, $k=i$

$$\triangledown \times \triangledown f=\varepsilon _{ijk}\partial _{j}f^{k}=0$$

because the three even terms cancel the three odd terms; the terms where two indices are equal are zero.

$$\triangledown .(\triangledown\times f)=\partial _{i}\varepsilon _{ijk}\partial _{j}f^{k}=\varepsilon _{ijk}\partial _{i}\partial _{j}f^{k}=0$$

By the same argument, the three terms of the even permutations of the Levi-Civita symbol cancel the three odd terms, and the rest are zero.
